# Vitamin D as a Systemic Regulatory Axis: From Homeostasis to Multiorgan Disease

**DOI:** 10.3390/biomedicines13112733

**Published:** 2025-11-07

**Authors:** María Rodríguez-Rivero, Miguel Ángel Medina

**Affiliations:** 1 Departamento de Biología Molecular y Bioquímica, Facultad de Ciencias, Andalucía Tech, Universidad de Málaga, 29071 Málaga, Spain; mar28042002@gmail.com; 2 IBIMA Plataforma BIONAND (Biomedical Research Institute of Málaga and Nanomedicine Platform), 29590 Málaga, Spain; 3 CIBER de Enfermedades Raras (Spanish Center of Researcher in Rare Diseases—CIBERER), 28029 Madrid, Spain

**Keywords:** vitamin D, health, disease, homeostasis, imbalance, supplementation, serum levels

## Abstract

**Background/Objectives**: To critically evaluate the current scientific literature on the physiological and preventive functions of vitamin D, with special emphasis on its possible involvement in multi-organ pathologies, and to assess the effectiveness of supplementation strategies for maintaining homeostasis. **Methods**: A review of the literature was conducted following a methodological approach in accordance with the PRISMA 2020 statement for systematic reviews. The bibliographic search was carried out in the PubMed, Scopus, and Web of Science databases, using controlled terms and Boolean operators. Rigorous inclusion and exclusion criteria were applied in three phases: blind search, selection by title/abstract, and full-text evaluation. Articles published in first quartile journals (JCR 2023) were prioritized. The search was complemented with targeted strategies such as consulting ORCID profiles, using the Jábega tool, and tracking cross-references. **Results**: The selected studies reinforce that vitamin D acts as a transcriptional modulator with effects beyond the skeletal system, including immunomodulatory, neuroprotective, and antitumor functions. Associations were identified between low levels of 25(OH)D and a higher prevalence of autoimmune, neurodegenerative, and metabolic diseases, as well as certain types of cancer. However, evidence of causality is still limited, and clinical trials have shown mixed results regarding its preventive efficacy. Supplementation strategies are useful in vulnerable populations, although their indiscriminate use without a documented deficiency is not recommended. **Conclusions**: Vitamin D is emerging as a potentially relevant agent in preventive medicine. While its benefits extrapolated from bone metabolism still require robust clinical validation, current findings support its role in regulating key systemic functions. A balanced approach combining sun protection, health education, food fortification, and targeted supplementation, tailored to the clinical context of each individual, is recommended.

## 1. Introduction

Within the framework of a renewed approach to medicine focused on health promotion, the integrity of barriers has been identified as the first hallmark of health, within the category of spatial compartmentalization [1,2]. Living organisms maintain their internal order through selective barriers that reduce entropy, with the skin being the most important of these. In addition to its protective function, the skin participates in the synthesis of vitamin D, a steroid hormone that is key to physiological balance.

### 1.1. Chemical and Biochemical Nature of Vitamin D

**Figure 1:**
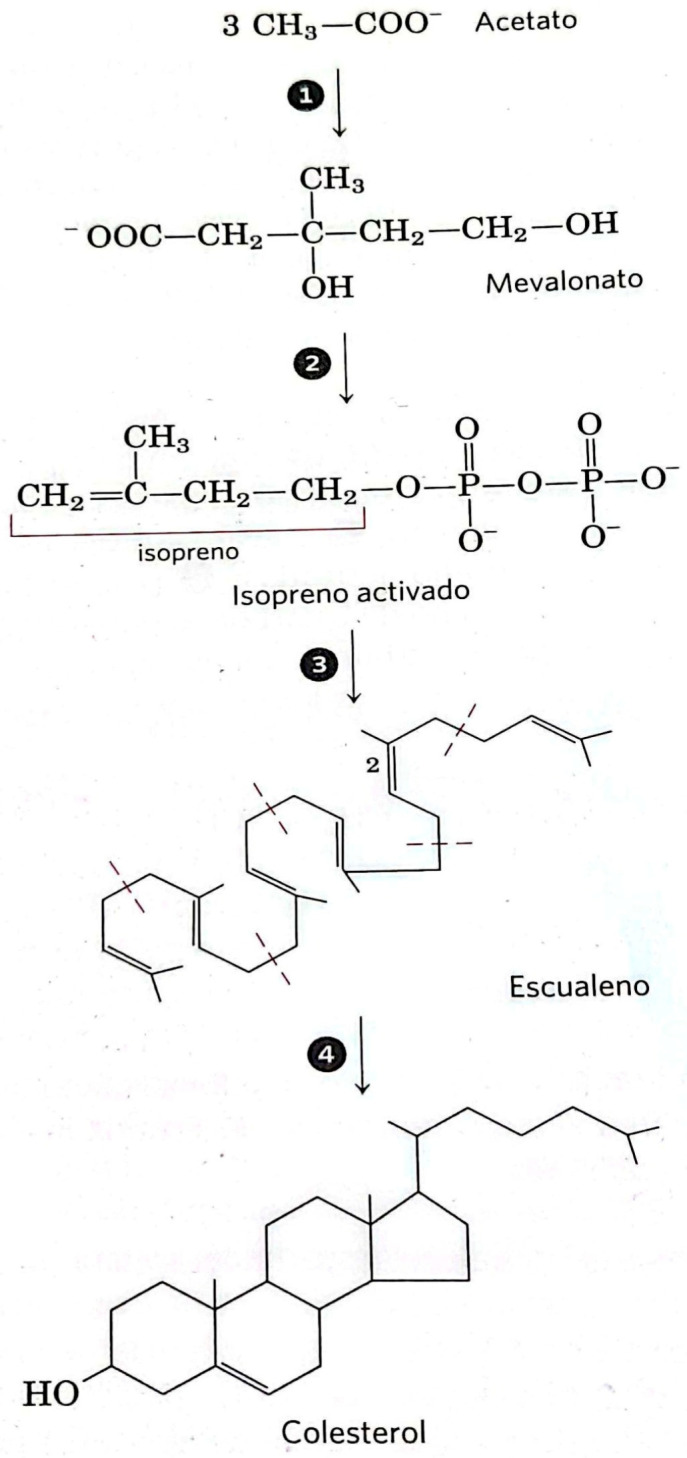
The four stages of cholesterol biosynthesis.

Vitamins are essential organic compounds that the human body cannot synthesize in sufficient quantities and must therefore be obtained from the environment. This definition, based on our metabolic limitations, underscores the indispensable nature of these substances for normal physiological functioning [3]. Vitamin D is a partial exception within this group, as the human body can synthesize it endogenously from cholesterol precursors, provided there is sufficient exposure to ultraviolet B (UVB) radiation. This characteristic places it in a unique position, halfway between an essential nutrient and a secosteroid hormone [4]. From a structural point of view, vitamin D is derived from cholesterol, whose synthesis begins with acetyl-CoA through a complex pathway divided into four phases (Figure 1). The generation of mevalonate marks the rate-limiting step of this process, mediated by the enzyme hydrixymethylglutaryl-CoA reductase, whose activity is regulated by both short-term metabolic signals and long-term transcriptional mechanisms in response to intracellular cholesterol levels [5]. Following the formation of squalene and subsequent cyclization to form the steroid nucleus, 7-dehydrocholesterol is generated, a key intermediate located in the epidermis. When exposed to UVB radiation, this compound undergoes photolysis, resulting in pre-vitamin D3, which is thermally converted to cholecalciferol (vitamin D3) at body temperature [4].

**Figure 2:**
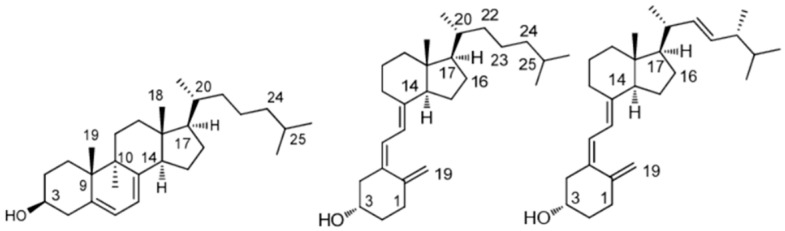
Chemical structures of 7-dehydrocholesterol (left), cholecalciferol (middle) and ergocalciferol (right).

Vitamin D comes in two main forms depending on its origin: ergocalciferol (vitamin D2), which comes from plant sources and fungi, and cholecalciferol (vitamin D3), which comes from animal or skin sources (Figure 2). Although they share metabolic pathways, they differ structurally due to the presence of an additional double bond and a methyl group in D2, which affects its affinity for transport proteins and the VDR, reducing its biological efficacy. For this reason, D3 is the preferred form in supplements and treatments [4]. Henceforth, and given the above, reference will be made mainly to vitamin D in general, understood as the D3 form unless otherwise indicated. It should be noted that none of these forms is active per se, but rather they require hepatic and renal transformation to exert their physiological functions through the VDR, which regulates genes involved in multiple processes beyond bone metabolism [6].

The hybrid nature of vitamin D and its dependence on UV radiation for synthesis determine its availability and justify the implementation of complementary strategies to ensure adequate intake.

### 1.2. Sources of Vitamin D: Challenges and Strategies for the General Population and Vulnerable Groups

Vitamin D is essential for bone health and other physiological functions. Its main source is its synthesis in the skin induced by UVB radiation. However, this represents a minimal fraction of the solar spectrum, and excessive exposure to ultraviolet radiation, especially UVA, is associated with risks such as skin aging, genetic damage, and skin cancer [7,8]. Every year, more than 60,000 deaths are linked to this exposure, mainly due to melanoma [9]. For this reason, the WHO advises against prolonged sun exposure and promotes protective measures through campaigns such as INTERSUN [10].

Given these limitations, food fortification and supplementation are effective and safe strategies, especially in areas with low solar radiation or among older people, whose skin synthesis capacity is reduced [11,12,13]. They are also essential in patients with malabsorptive gastrointestinal diseases, in whom vitamin D deficiency can aggravate the clinical course of their pathologies [14]. In these cases, it is recommended to maintain serum 25(OH)D levels above 50 nmol/L.

While the correction of severe deficiencies is indisputable, the extraosseous benefits of vitamin D have not yet been clearly demonstrated [15]. Therefore, widespread supplementation in healthy individuals without documented deficiency is not justified, and it is preferable to focus interventions on risk groups [16,17].

A balanced approach should combine sun protection, education, food fortification, and targeted supplementation, always based on clinical criteria, to prevent hypovitaminosis D without incurring unnecessary risks.

### 1.3. Justification for This Work

Vitamin D is a unique nutrient whose synthesis depends on sun exposure and individual factors, which can lead to deficiencies in certain groups. Although its role in bone health is well established, uncertainties remain about its possible systemic effects and extraosseous benefits.

This study aims to critically review the current scientific literature, investigating those functions that are not yet clearly defined, and to provide a solid basis for guiding clinical and public health decisions, especially in a context where its diagnostic determination has been restricted in some regions.

## 2. Review Methodology

It was decided to carry out a scientific review according to the guidelines provided by PRISMA 2020 statement for systematic reviews [18]. Three databases were used: PubMed (https://www.ncbi.nlm.nih.gov/), Scopus (https://www.scopus.com), and Web of Science (WOS) (https://www.webofscience.com). In addition to using bibliographic databases, ORCID (Open Researcher and Contributor ID) was used, an author identification system through which it was possible to locate the profile of the researcher Alberto Muñoz (https://orcid.org/0000-0003-3890-4251) and access his scientific output. This allowed for a parallel, more targeted search for relevant articles in the field of cancer. Likewise, the Jábega tool (http://jabega.uma.es/), provided by the University of Malaga, was used to locate general handbooks and other useful documentary resources in the preparation of Section 1.

### Workflow and Phases of the Literature Search

**Figure 3:**
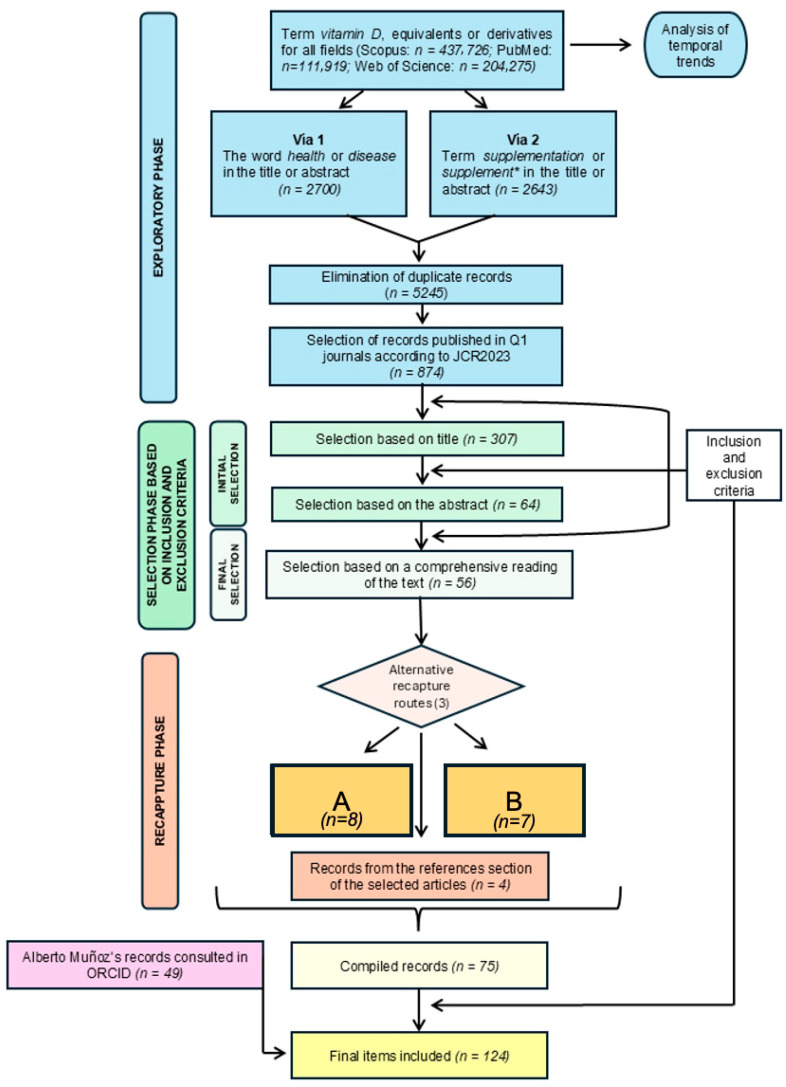
Workflow chart of the review methodology. The numbers of records selected in each stage are also shown. A: Records prior to the search in scientific literature databases. B: Records obtained/published after finishing the search in scientific literature databases.

The procedure used for the literature search was organized into three clearly differentiated phases, represented in the workflow chart by different colors (Figure 3).

First, there was an exploratory phase, also known as blind search, in which an initial review is carried out without applying previously defined criteria. Next, a second phase was carried out, focusing on the selection of records according to previously established inclusion and exclusion criteria. This stage, in turn, is divided into two sub-phases: an initial selection, based on a review of the title and abstract of the articles, and a final selection, in which the criteria are applied to the full text of the previously selected documents. Finally, a third phase was carried out, corresponding to the recapture of information. In this stage, new references were incorporated through complementary channels external to the formal search process. These channels include records published after finishing the exploratory phase, documents that had been selected before the start of the work, and articles cited in the bibliographic reference sections of the works selected at the end of the second phase. In addition to these phases, complementary search strategies were used to identify key authors in the area of study. In particular, the ORCID profile of the researcher Alberto Muñoz, a first level expert in vitamin D and cancer, was consulted, which made it possible to select publications that were particularly relevant to this work.

In the first phase of the search process or blind search phase, a series of restrictive criteria were applied, as detailed in the specific syntax used in each database, as detailed in Supplementary Materials File S1. The databases offered additional filters, such as delimitation by year of publication; however, this filter was not used. The decision was based on the risk of excluding classic articles of great relevance which, although old, remain fundamental to understanding and explaining the development of the topic. Initially, a combination of keywords equivalent to or derived from the term “vitamin D” was entered in all search fields (in Web of Science, it was not possible to perform a blind search for all fields, but only according to topic). Subsequently, the search was restricted so that these terms appeared specifically in the title, and other general words were delimited to appear in the title or abstract. From this point on, the strategy branched into two paths: the first, focused on studies on vitamin D in contexts of balance (health) and imbalance (disease); and the second, focused on research addressing supplementation. Numerous duplicate records were identified. Therefore, all articles found in pathway 1 were first merged through PubMed, Scopus, and Web of Science (WOS), and the same process was carried out independently with the records from pathway 2. Next, after removing the duplicates in each pathway, a new merge was performed combining both pathways, due to the existence of additional duplicates between them. After removing documents with duplicate titles, those published in first quartile journals according to the Journal Citation Reports 2023 (JCR2023) were selected.

With the database of records already refined, the selection phase was carried out by applying the inclusion and exclusion criterio for titles (Table 1), abstracts (Table 2) and full texts (Table 3). The exploratory and selection phases of our search were carried out from October 2024 to January 2025. The recapture phase remained open up to the initial submission date of this article (11 September 2025).

## 3. Results of the Bibliographic Search

**Figure 4:**
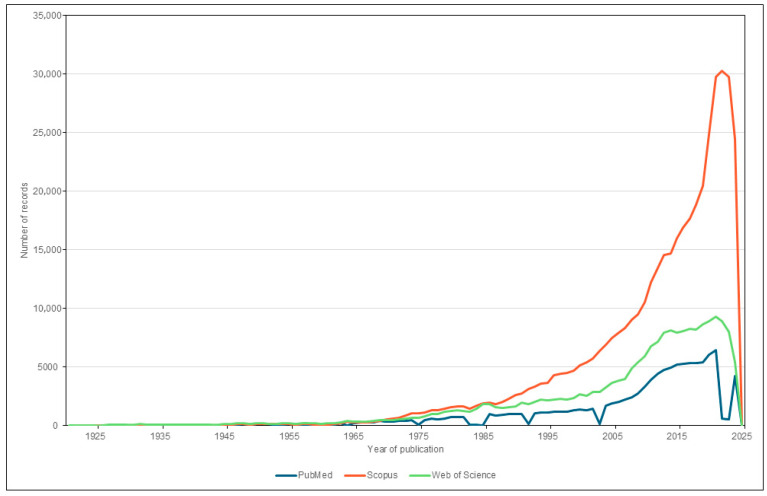
Curves showing the accumulated number of records with time of publications on vitamin D stores in PubMed, Scopus and Web of Science.

The analysis of temporal trends for the records initially obtained in each database used at the beginning of the exploratory phase is shown in Figure 4. It is clear that the curves begin to grow visibly at the beginning of the 21st century, increasing significantly in slope around 2013, until reaching their current peak in approximately 2023.

The fluctuations observed in the PubMed graph correspond to the changes that the database itself has undergone throughout its history. It should be noted that PubMed emerged in the mid-20th century, when the US National Library of Medicine began digitizing medical publications that had been published since 1879, leading to the creation of MEDLARS (MEDical Literature Analysis and Retrieval System) in 1966. For this reason, the graph shows how this exponential growth began around the year 1965. Subsequently, the application of CD-ROM in the 1980s led to a temporary decline in publications, which allowed for the subsequent use of this format in the database and definitively launched MEDLINE (MEDlars onLINE). Thus, changes have gradually been made in line with the technological advances of each era which, despite having led to pauses in publications, have subsequently allowed for the establishment of more user-friendly formats that facilitate the search for bibliographic information [19].

## 4. Metabolism and Physiopathological Functions of Vitamin D

**Figure 5:**
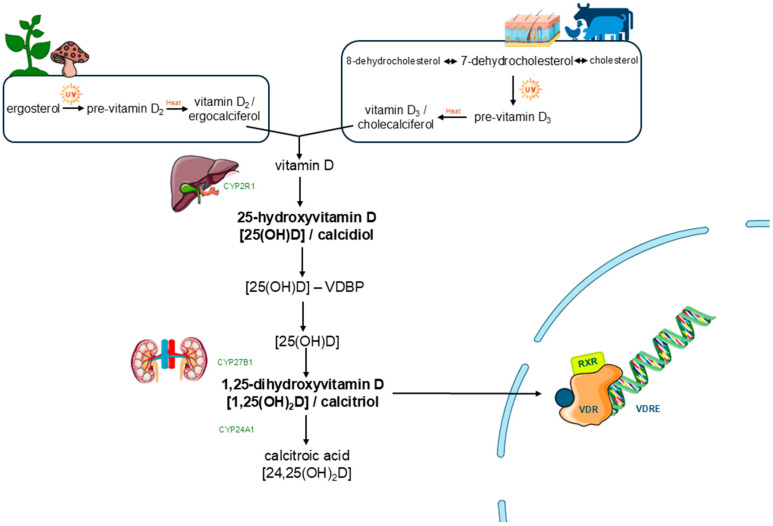
Metabolism and action mechanism of vitamin D. Some icons have been obtained form Servier Medical Art, with licence Creative Commons Attribution 4.0 (https://smart.servier.com).

Vitamin D3 (cholecalciferol) is first hydroxylated in the liver to form 25-hydroxyvitamin D [25(OH)D] by the action of CYP2R1 (the most relevant in humans) and then in the kidney by the enzyme 1α-hydroxylase (CYP27B1) into the active metabolite 1,25-dihydroxyvitamin D3 [1,25(OH)_2_D_3_] or calcitriol, which acts as a hormone in the cell nucleus of various target cells via the vitamin D receptor (VDR), as shown in Figure 5 [20,21,22]. The vitamin D receptor (VDR) belongs to the nuclear receptor superfamily, which shares a modular structural organization characterized by a DNA-binding domain in the central region and a ligand-binding domain (LBD) at the carboxyl terminus. The latter is essential for calcitriol binding and for the transmission of its signal at the transcriptional level. The LBD has a conserved structural conformation, composed of a set of helices that form a sandwich-type core, where a specific hydrophobic pocket for the ligand is located. The binding of calcitriol induces a conformational change in helix 12 (H12), also known as AF-2, which facilitates the recruitment of coactivators (such as retinoid X receptor, RXR) and the initiation of gene transcription [23,24].

**Figure 6:**
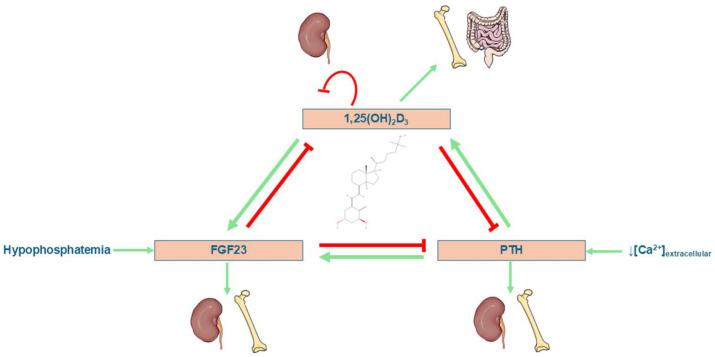
Diagram of the hormonal interactions between PTH, FGF23, and 1,25(OH)_2_D_3_ (calcitriol) in the regulation of calcium and phosphate. Some icons have been obtained form Servier Medical Art, with licence Creative Commons Attribution 4.0 (https://smart.servier.com).

Vitamin D metabolism is subject to precise control that allows calcitriol production to be adjusted according to the body’s physiological needs. Calcitriol synthesis is regulated by feedback mechanisms involving calcium, phosphorus, and parathyroid hormone (PTH), as documented in classic studies [25]. In a state of hypocalcemia, PTH secretion stimulates CYP27B1 activity, promoting calcitriol production (Figure 6). In addition, hypophosphatemia also stimulates this enzyme, even in the absence of PTH [26,27,28]. The involvement of G protein-coupled receptors and the Scribble protein in the modulation of phosphocalcic and vitamin D metabolism has also been described [29]. Severe alterations in mineral metabolism have been observed in murine knockout models for the enzyme 1α-hydroxylase, which can be partially corrected with calcium-rich diets, regulating renal calcium transport genes [30]. In addition, the SDR42E1 gene has been identified as a regulator of vitamin D synthesis through the control of steroid biosynthesis and key precursors such as 7-dehydrocholesterol (see Figure 5) [31]. Taken together, these findings demonstrate the complexity of the mechanisms that regulate the availability of active vitamin D in the body.

Among its many functions, the active form of vitamin D stands out for its central role in the body’s mineral balance. Calcitriol is crucial for calcium and phosphorus homeostasis, promoting their intestinal absorption, renal reabsorption, and bone mineralization (see Figure 6) [32]. The discovery of vitamin D and these effects led to the historic eradication of rickets [33,34]. In a study by Bikle et al. in humans [35], a correlation was demonstrated between calcitriol levels and intestinal alkaline phosphatase activity, supporting its role in intestinal adaptation to dietary calcium. The absence of the vitamin D receptor (VDR) in mice (after weaning) leads to a severe phenotype characterized by hypocalcemia, rickets, alopecia, growth retardation, and reproductive disorders, indicating the essential role of the vitamin D-VDR axis [36]. These findings reinforce the importance of vitamin D in bone and mineral physiology from early stages of development. Likewise, it has been shown that, in contexts of metabolic stress, such as severe thermal injuries, vitamin D not only prevents the loss of bone mineral density, but also contributes to restoring cellular energy metabolism. In a murine burn model, vitamin D administration improved Krebs cycle metabolite levels in muscle, increased the availability of key amino acids such as alanine and glutamine, and reversed the loss of mesenchymal stem cells, reinforcing its protective role against bone and tissue catabolism in critical situations [37].

Calcitriol, the active form of vitamin D, together with its nuclear receptor VDR, plays a key role in regulating genes involved in various pathologies, especially inflammatory, autoimmune, and oncologic diseases. VDR acts as a transcriptional regulator whose activity has been extensively studied using techniques such as ChIP-seq, which have identified thousands of binding sites throughout the human genome, many of them related to autoimmune diseases and cancer [38]. In addition, certain transcriptomic studies have shown that other vitamin D metabolites, such as 25(OH)D_3_ and 24R,25(OH)_2_D_3_, also exert regulatory effects on genes linked to non-classical functions, such as cell differentiation and immunomodulation [39]. Using mathematical models, it has been shown that the bioavailability of vitamin D in its free active form is conditioned by its affinity for the transport protein (VDBP), which directly influences the intensity of the cellular response [40]. This systemic regulation extends to other metabolic axes, such as iron, where vitamin D modulates the expression of hepcidin, opening up new therapeutic possibilities for the treatment of anemia [41].

From a population perspective, various epidemiological studies have identified inverse associations between serum vitamin D levels and the prevalence of chronic diseases such as cancer, cardiovascular disease, and type II diabetes. However, although these associations suggest a protective effect, randomized clinical trials have not yet provided conclusive results on its therapeutic efficacy [42].

At the cellular and molecular level, vitamin D-mediated signaling does not act in isolation but is integrated into complex networks that modulate key functions in both physiological and pathological states. Calcitriol can interact with other intracellular pathways, such as those of EGFR (epidermal growth factor receptor) and PDGFR (platelet-derived growth factor receptor). For example, in combination with EGF, it enhances the expression of genes such as CYP24A1, while PDGF-BB generates different responses without directly altering VDR activity [43]. Vitamin D also regulates other pathways such as Wnt and TGF-β, which are linked to the control of cell proliferation, differentiation, and paracrine communication [44]. These molecular interactions help explain its role in inhibiting processes such as epithelial–mesenchymal transition (EMT), which is relevant in tumor progression, fibrosis, and embryonic development. Calcitriol induces cell adhesion genes and suppresses pro-EMT factors, which in turn can decrease VDR expression, generating a reciprocal regulatory loop [45]. In this context, the interaction of the PTH receptor with proteins such as Scribble links mineral metabolism with cell polarity, an aspect that is also affected during EMT [29]. The inhibitory effect of calcitriol on cell proliferation also extends to its ability to promote cell differentiation and modulate the phenotype of tumor-associated fibroblasts and cancer stem cells through the inhibition of pathways such as Wnt/β-catenin, TGF-β, and EGF [46]. Calcitriol modulates gene expression by acting on both the epigenome and the transcriptome, reinforcing its therapeutic potential in oncology by regulating processes such as cell differentiation, proliferation, and immunomodulation [47]. This consolidates the inhibitory role of vitamin D on EMT, with implications for tumor pathophysiology. Taken together, these findings demonstrate that the effects of vitamin D on human health transcend the control of bone metabolism, integrating into complex signaling networks that could explain its involvement in multiple chronic diseases. The function of this metabolite as a transcriptional modulator and cellular signaling molecule constitutes the molecular basis for its systemic effects observed at the clinical and epidemiological levels.

## 5. The Role of Vitamin D in Multi-Organ Pathologies

**Figure 7:**
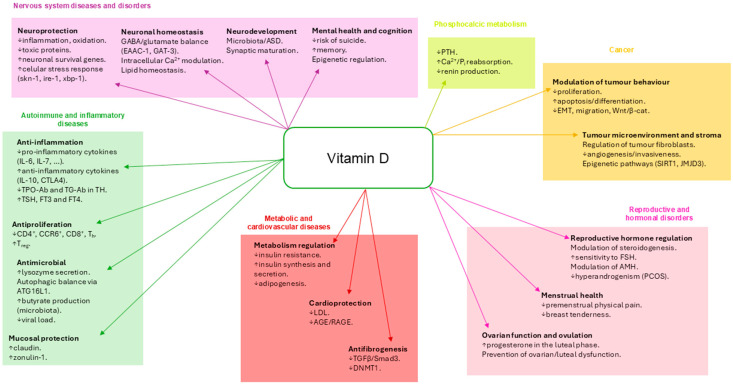
Systemic involvement of vitamin D in health and disease.

The widespread distribution of the vitamin D receptor (VDR) in different tissues and cells suggests a functional role beyond bone metabolism, with implications for systems such as the immune, nervous, and cardiovascular systems, as well as metabolic and hormonal processes. In this context, Figure 7 visually summarizes the involvement of vitamin D in different pathophysiological spheres. This integrative view reinforces the concept of vitamin D as a systemic modulator with great potential in the field of preventive and personalized medicine. This section addresses the main experimental and clinical studies linking alterations in the vitamin D–VDR axis to various non-skeletal and non-tumoral diseases. Section 6 will be devoted to the role of vitamin D in cancer.

### 5.1. Autoimmune and Inflammatory Diseases

Vitamin D plays a key role in regulating the immune system, influencing the pathogenesis and possible treatment of various autoimmune and inflammatory diseases [48]. In diseases such as multiple sclerosis (MS), an association has been observed between low vitamin D levels and increased susceptibility to the disease [49]. This effect is related to vitamin D’s ability to modulate the response of T cells, inhibiting their proliferation—including myelin peptide-specific CD4^+^ cells—and modifying the cytokine profile by reducing IL-6 and IL-17 and increasing IL-10 production. In animal models with experimental autoimmune encephalomyelitis (EAE), the administration of calcitriol has been shown to prevent the onset of the disease, even in the absence of CD8^+^ T cells, suggesting an action on other immune subpopulations [49,50]. Clinically, in patients with relapsing-remitting MS, higher levels of 25(OH)D have been associated with a lower relapse rate, showing a 27% reduction in risk for each doubling of serum concentrations, suggesting a dose-dependent effect [51].

This immunomodulatory effect is part of a broader context in which vitamin D not only acts as an environmental protective factor against multiple sclerosis, but also interacts directly with the HLA-DRB1*1501 allele, the main genetic factor involved in susceptibility to the disease. This functional relationship reinforces the idea that the development of multiple sclerosis results from the convergence of hereditary predisposition and environmental conditions [52,53].

In other immune-mediated diseases, such as rheumatoid arthritis (RA), the active metabolite calcitriol reduces the proinflammatory profile of CCR6^+^ memory T helper cells—producers of IL-17A, IFN-γ, and TNF-α—and induces the expression of IL-10 and CTLA4, giving them regulatory properties without affecting their migratory capacity to inflammatory environments such as synovial fluid [54]. In atopic dermatitis (AD), the expression of the vitamin D receptor (VDR) in keratinocytes and immune cells indicates that its activation contributes to the control of skin immunity and the maintenance of epidermal homeostasis [55]. In psoriasis, the usual antiproliferative action of calcitriol appears to be altered. Although calcitriol normally inhibits fibroblast proliferation, an increase in c-MYC (a proto-oncogene associated with cell proliferation) is observed in psoriatic cells, suggesting a possible dysfunction in the way these cells respond to vitamin D metabolism [56].

In the field of inflammatory bowel diseases (IBD), such as ulcerative colitis and Crohn’s disease, the interaction between vitamin D and its receptor is essential for maintaining intestinal immune homeostasis. Epithelial VDR dysfunction is associated with imbalances in the microbiome, reduced antimicrobial mechanisms such as lysozyme production by Paneth cells, and impaired ATG16L1-mediated autophagy [57]. This alteration compromises immune tolerance and may perpetuate chronic inflammation. In murine models, vitamin D deficiency decreases colonic mucosal thickness, elevates proinflammatory cytokines, and reduces tight junction proteins such as claudins and zonulin-1, affecting the integrity of the intestinal barrier [58]. In addition, chronic vitamin D deficiency can affect other organs such as the kidneys, promoting microvascular dysfunction and activation of the renin-angiotensin system, which, together with dietary factors such as high fructose consumption, aggravates vitamin deficiency [59,60]. On the other hand, metabolites such as butyrate, produced by the microbiota, regulate the expression of VDR and ATG16L1, establishing a feedback loop relevant to intestinal immunoregulation [57]. This relationship could also extend to other organs exposed to the microbial environment, such as the skin or lungs.

In relation to lung conditions, low levels of 25(OH)D (<20 ng/mL) have been associated with poorer respiratory function and greater clinical uncontrolledness in asthmatic patients [61]. During SARS-CoV-2 infection, vitamin D deficiency has been linked to increased clinical severity and phenomena such as cytokine storm, suggesting that supplementation could be beneficial in vulnerable populations [62]. Complementarily, preclinical studies have shown that VDR agonists can reduce the viral load of dengue virus when administered after infection, which broadens their potential therapeutic utility [63].

In Hashimoto’s thyroiditis (HT), the leading cause of hypothyroidism in regions with adequate iodine intake, vitamin D also shows beneficial effects. This autoimmune disease is characterized by chronic inflammation and progressive destruction of the thyroid gland, with the presence of autoantibodies such as TPO-Ab and TG-Ab. A recent meta-analysis showed that vitamin D supplementation significantly reduces these autoantibodies, improves TSH, FT3, and FT4 levels, and exerts immunoregulatory effects by modulating Th and Treg cells, decreasing proinflammatory cytokines, and promoting anti-inflammatory mediators. These benefits were more noticeable with active vitamin D and treatments lasting longer than 12 weeks, supporting its complementary use in HT, although further studies are needed to define optimal guidelines [64].

Taken together, these findings reinforce the role of vitamin D as a cross-sectional immune modulator, capable of influencing multiple diseases through the vitamin D/VDR axis. Its impact on the integrity of epithelial barriers, inflammatory response, and immune tolerance positions it as a key player in the study and treatment of autoimmune, inflammatory, and infectious diseases.

### 5.2. Metabolic and Cardiovascular Diseases

Vitamin D plays an important role in regulating metabolic and cardiovascular processes. Its plasma levels have been found to correlate with the risk of developing diseases such as diabetes, dyslipidemia, non-alcoholic fatty liver disease (NAFLD), and cardiovascular pathologies.

In relation to carbohydrate metabolism, several studies have identified an inverse association between 25-hydroxyvitamin D concentration and insulin resistance, especially in non-Hispanic white and Mexican populations, but not in African Americans, suggesting possible ethnic variability in vitamin D sensitivity [65,66]. This relationship has also been addressed from a molecular perspective, showing that vitamin D can modulate the expression of anti- and proinflammatory cytokines, Toll-like receptors, and pathways related to insulin sensitivity [67].

During early development, maternal vitamin D deficiency, combined with a high-fat diet, negatively affects the glucose homeostasis of offspring, with a greater impact on males, by altering the transcriptomic profiles of adipose tissue [68]. Complementarily, studies in zebrafish have shown that prolonged deficiency generates an altered metabolic phenotype, with central adiposity, dyslipidemia, and alterations in insulin and growth hormone signaling [69].

In lipid homeostasis, an inverse relationship between vitamin D and LDL has been observed, suggesting a potential protective effect against dyslipidemia and cardiovascular disease [70]. At the hepatic level, active vitamin D supplementation reduces the progression of NAFLD by modulating the p53 pathway, decreasing lipogenesis, apoptosis, senescence, and hepatic oxidative stress [71]. In addition, it exerts beneficial epigenetic effects in NAFLD by inhibiting DNMT1 and regulating the TGFβ1/Smad3 pathway, which attenuates fibrosis [72].

In chronic kidney disease, high doses of vitamin D3 reduce fibrosis and epithelial–mesenchymal transition by activating VDR and inhibiting the TGF-β1/Smad3 pathway [73]. It also improves innate immunity in hemodialysis patients by increasing 25(OH)D levels and reducing proinflammatory cytokines such as IL-6, IL-8, and TNF in monocytes through VDR-dependent proteins [74].

In the context of chronic alcohol-induced liver damage, vitamin D deficiency aggravates the disease by altering the gut microbiome, compromising the epithelial barrier, and activating the NF-κB pathway, which increases liver inflammation [75].

Finally, in the cardiovascular sphere, vitamin D acts on the AGE/RAGE axis, which is involved in arterial stiffness, mitochondrial dysfunction, and oxidative stress, establishing itself as a potential therapeutic target [76].

Taken together, these findings support the integral role of vitamin D in metabolic balance, immune regulation, and liver and cardiovascular protection, underscoring the importance of maintaining adequate levels of vitamin D throughout life.

### 5.3. Nervous System Dysregulation and Diseases

Vitamin D has been identified as a neurosteroid capable of exerting genomic and non-genomic effects on the central nervous system. Its deficiency has been linked to various neurodegenerative diseases, neurodevelopmental disorders, and mental health disorders. Its neuroprotective action is based on multifactorial mechanisms, such as controlling intracellular calcium homeostasis, regulating neuroinflammation, reducing oxidative stress, and modulating genes involved in neuronal survival. In addition, it has been observed that it can interfere with pathological processes such as the aggregation of neurotoxic proteins characteristic of Alzheimer’s and Parkinson’s diseases [77].

Certain experimental studies have delved into its role in protein homeostasis and longevity. In *Caenorhabditis elegans*, vitamin D3 administration extended life expectancy by activating stress response pathways mediated by the skn-1, ire-1, and xbp-1 genes, reducing age-related protein insolubility, and protecting against human β-amyloid toxicity [78]. These results point to a direct role for vitamin D in preventing neurodegenerative decline.

During critical stages of development, such as puberty, hypovitaminosis D affects cortical presynaptic functionality. In animal models, a reduction in glutamate and GABA uptake and release has been observed, along with alterations in EAAC-1 and GAT-3 transporters, compromising the excitatory-inhibitory balance and the brain’s response to hypoxic episodes [79].

The control of oxidative stress represents another relevant axis. In a model of LPS-induced cognitive impairment in rats, vitamin D3 administration improved memory and learning, possibly by inhibiting IL-6 and increasing the activity of antioxidant enzymes such as catalase and superoxide dismutase in the hippocampus [80].

A link between vitamin D deficiency and alterations in brain lipid metabolism has also been described. In cellular and murine models, this deficiency caused changes in phosphatidylcholines, plasmalogens, and carnitines, which are essential for neuronal membrane integrity and energy homeostasis, potentially contributing to neurological pathologies associated with lipid dysfunction [81].

Supplementation with vitamin D, together with folic acid and vitamin B12, reversed the cognitive impairment associated with its deficiency. This effect was related to the modulation of the CYP27A1 gene and the regulation of 27-hydroxy cholesterol, which is involved in brain cholesterol metabolism and the pathogenesis of Alzheimer’s disease. Likewise, variations in homocysteine and S-adenosylmethionine levels were observed, suggesting an interaction between lipids, vitamins, and neurotransmitters [82].

In the field of mental health, certain studies have found an association between serum vitamin D levels and suicidal behavior. In a cohort of US veterans, supplementation with vitamin D2 and D3 was associated with a significant reduction in the risk of suicide and self-harm, especially in people with low vitamin D levels (<20 ng/mL) and in the African American population, showing a dose-dependent effect [83].

With regard to neurodevelopmental disorders, such as autism spectrum disorder (ASD), the results are less conclusive. A Mendelian randomization analysis found no evidence of a direct causal relationship between vitamin D levels and the risk of ASD, although individuals with ASD were found to have lower serum levels, suggesting a possible inverse association [84]. In mouse models, vitamin D deficiency during pregnancy and lactation altered the gut microbiota of offspring, inducing repetitive behaviors and social deficits characteristic of ASD, effects that were reversed by fecal transplantation, highlighting the role of the gut–brain axis in neurodevelopment [85].

Taken together, the evidence reinforces the fundamental role of vitamin D in the health of the nervous system, both in its development and in its functional maintenance throughout life. Its deficiency can affect multiple levels—molecular, cellular, and systemic—compromising processes such as neurotransmission, inflammation, lipid and protein metabolism, as well as emotional state. In this context, its assessment and supplementation, especially in vulnerable populations, could constitute a preventive or complementary strategy in addressing neurological and neuropsychiatric pathologies.

### 5.4. Reproductive and Hormonal Disorders

Vitamin D has been linked to various aspects of female reproductive physiology, acting on tissues such as the ovaries, endometrium, and placenta, where its receptors are expressed, suggesting a regulatory role in key hormonal processes. A systematic review highlights its influence on steroidogenesis, FSH sensitivity, progesterone production, and AMH signaling. In women with polycystic ovary syndrome (PCOS), supplementation has shown benefits such as reduced AMH and a possible anti-inflammatory effect, although its impact on pregnancy rates after IVF remains uncertain [86]. Observational studies have associated low levels of 25(OH)D with hormonal disturbances, such as elevated levels of LH, testosterone, AMH, and FAI, which could promote a hyperandrogenic environment characteristic of PCOS [87].

In cases of unexplained infertility, a relationship has also been observed between vitamin D deficiency and lower progesterone production in the luteal phase, suggesting a possible involvement in ovulation or corpus luteum function [88].

On the other hand, insufficient vitamin D levels have been linked to premenstrual physical symptoms, such as generalized pain and breast tenderness, unrelated to psychological symptoms or other markers of calcium metabolism [89].

Taken together, this evidence suggests that vitamin D deficiency may contribute to hormonal imbalances, ovulatory dysfunction, and premenstrual symptoms. Although further clinical studies are needed, its assessment may be relevant in the management of female reproductive disorders.

## 6. The Role of Vitamin D in Cancer

Vitamin D has been extensively researched for its role in cancer prevention and modulation. In vitro, in vivo, and epidemiological studies support its antitumor action through genomic and non-genomic mechanisms that affect proliferation, apoptosis, differentiation, angiogenesis, and interaction with the tumor microenvironment [90].

The active form of vitamin D, calcitriol, binds to its nuclear receptor (VDR) and regulates the transcription of numerous genes involved in cell cycle control, differentiation, and tumor suppression [91]. At the post-transcriptional level, it also modulates the expression of proteases and their inhibitors, such as cystatin D, with relevant effects on tumor biology [92].

Several studies have shown that low levels of 25(OH)D are associated with lower survival rates in cancer patients, with a dose–response relationship and possible causality based on epidemiological criteria such as temporality and consistency of results. Although possible biases have been pointed out, most research suggests that vitamin D deficiency could aggravate the clinical course of cancer [93].

In the context of breast cancer, calcitriol has been shown to modify cellular architecture, induce the expression of integrins and keratins, and suppress myoepithelial and mesenchymal markers such as SMA and N-cadherin, reflecting a differential action on cellular identity and a possible anti-metastatic effect [94]. Furthermore, in this type of tumor, as well as in gliomas, vitamin D inhibits the expression of tenascin-C, an extracellular matrix protein involved in proliferation, invasiveness, and angiogenesis. This inhibition has been observed in both mammary epithelial cells and glioma cell lines, even blocking its induction by tumor-promoting agents, which reinforces the regulatory role of vitamin D in modulating the tumor microenvironment [95,96].

In urothelial bladder cancer, low levels of 25(OH)D_3_ correlate with an increased risk of developing muscle-invasive tumors, especially in cases with low FGFR3 expression. In cell cultures, calcitriol increases FGFR3 expression, suggesting a possible protective mechanism [97].

In relation to melanoma and other skin cancers, certain observational studies and meta-analyses indicate that vitamin D deficiency is associated with a worse prognosis, especially in terms of greater tumor thickness and poorer survival [98,99]. In fact, it has been suggested that low skin availability of vitamin D, rather than UVB radiation exposure itself, could be a relevant determinant in the progression of melanoma, while UVA radiation—which can penetrate windows and certain sunscreens—has been identified as a possible additional procarcinogenic factor [100]. The pleiotropic effects of vitamin D on the cell cycle, DNA repair, and apoptosis reinforce its potential role as a tumor suppressor in the skin [101].

It has been proposed that vitamin D may mitigate some procarcinogenic effects of obesity such as chronic inflammation, insulin resistance, or oxidative stress, although its action is limited due to the reduced bioavailability of the vitamin in obese individuals [102].

Several epidemiological and preclinical studies have linked low vitamin D levels to an increased risk of developing colorectal cancer. The active metabolite 1α,25-dihydroxyvitamin D_3_ acts through the vitamin D receptor to modulate numerous cellular processes, including proliferation, differentiation, apoptosis, and migration. These actions are mediated by genomic and non-genomic mechanisms, making vitamin D a multifunctional regulator with therapeutic potential in this type of neoplasm [103,104,105].

### 6.1. Molecular and Cellular Mechanisms Involved in Colon Cancer: Genomic Actions of Vitamin D Through VDR

Calcitriol exerts its direct transcriptional action on multiple genes involved in the tumor biology of colon cancer. Interference with the Wnt/β-catenin pathway (which is activated in almost all colon cancers) is one of its most notable effects. The active form of vitamin D directly interferes with this oncogenic pathway by inducing adhesion proteins such as E-cadherin, nuclear export of β-catenin, and repression of proto-oncogenes such as c-MYC, TCF1, and CD44 [106,107,108,109]. Likewise, calcitriol activates the expression of the DKK-1 gene, an extracellular Wnt inhibitor, and represses DKK-4, which promotes migration and angiogenesis in human colon cancer cells, thereby reinforcing its antitumor action [110,111,112]. This action has been confirmed in animal models, where the absence of VDR potentiates the activation of this pathway, exacerbating tumorigenesis induced by mutations in the APC tumor suppressor gene [113].

Other transcriptional effects include the modulation of cell cycle inhibitor and promoter genes such as Id1 and Id2, which promote epithelial differentiation and reduce proliferation [114]. Through transcriptomic and proteomic analyses, it has been shown that calcitriol broadly regulates the expression of genes involved in adhesion, intracellular signaling, proliferation, and oxidative stress. Target genes include c-JUN, NES-1, keratin-13, CST5 (cystatin D), and several epithelial differentiation factors [115,116].

In addition, vitamin D induces the expression of histone demethylase JMJD3, which enhances the regulation of epithelial genes mediated by VDR [117,118], thus integrating epigenetic regulation into its transcriptional action. It also promotes the expression of microRNA-22, which contributes to its antiproliferative and antimigratory effects through the post-transcriptional modulation of numerous genes involved in these functions [119].

In contrast to these beneficial effects, there are mechanisms that limit or block the gene action of vitamin D, especially in advanced stages of cancer. Various transcription factors such as SNAIL1 and SNAIL2, drivers of epithelial–mesenchymal transition (EMT), directly repress VDR expression, preventing the action of calcitriol in late stages of cancer. These factors additively suppress the transcription of VDR and E-cadherin (CDH1), inhibit the nuclear export of β-catenin, and inhibit the induction of genes such as CYP24A1 [120,121,122]. Their influence extends beyond tumor cells to histologically normal adjacent tissue, altering the expression of VDR and E-cadherin in areas surrounding the tumor [123].

The combination of the transcription factors SNAIL and ZEB1, together with the co-regulators p300 and CtBP, has been associated with the repression of the VDR and CDH1 genes, which are key in epithelial differentiation [124]. In fact, clinical studies in colorectal cancer have shown that SNAIL expression is associated with a decrease in VDR and CDH1, a correlation that is lost in the presence of high levels of ZEB1. Low expression of both genes is linked to more aggressive tumor characteristics, such as poor differentiation, vascular invasion, lymph node metastasis, and advanced stages [125].

### 6.2. Molecular and Cellular Mechanisms Involved in Colon Cancer: Activation of Non-Genomic Pathways of Vitamin D

**Figure 8:**
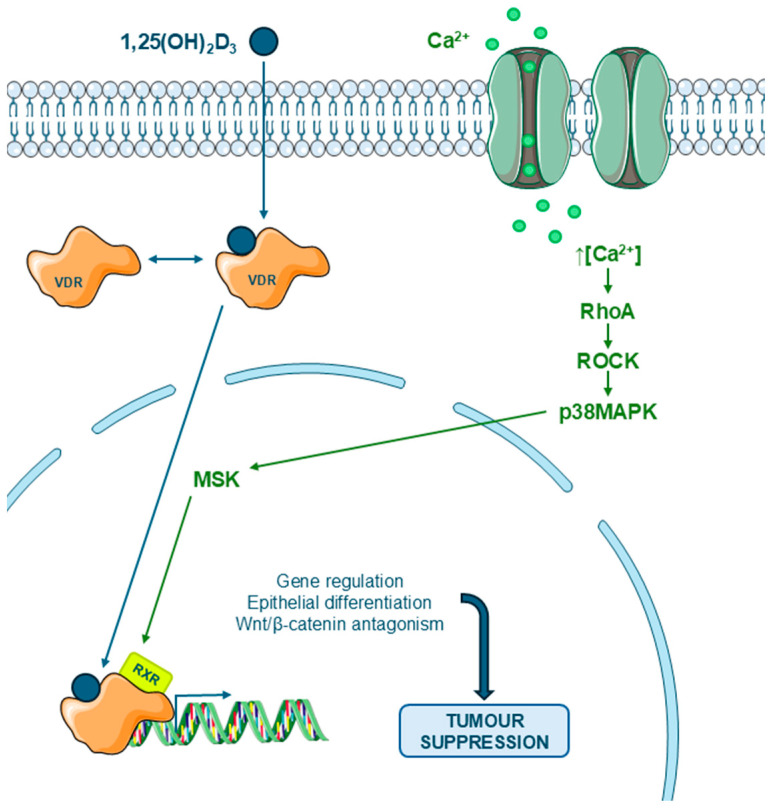
Diagram of the process by which 1,25(OH)_2_D_3_ (calcitriol) induces the activation of cellular signaling pathways (RhoA-ROCK and p38MAPK-MSK) in colon cancer cells, following an initial increase in cytosolic calcium, thus promoting the expression of suppressor genes. Some icons have been obtained form Servier Medical Art, with licence Creative Commons Attribution 4.0 (https://smart.servier.com).

In addition to its classic transcriptional action, calcitriol also exerts relevant non-genomic effects in colon cancer cells. Among these, the rapid and independent activation of transcription of the RhoA–ROCK–p38MAPK–MSK1 pathway, initiated by an increase in cytosolic calcium, stands out (Figure 8).

This pathway is necessary to induce epithelial differentiation, cell adhesion (through proteins such as E-cadherin, occludin, and vinculin), and the regulation of genes such as cyclin D1, CYP24A1, and CST5/cystatin D. Pharmacological or genetic inhibition of these components completely blocks vitamin D-induced epithelial morphology and its antiproliferative effects, including inhibition of the Wnt/β-catenin [126,127].

On the other hand, proteomic studies revealed that calcitriol regulates nuclear proteins associated with RNA processing, including SFPQ, SMARCE, KHSRP, TARDBP, and PARP1, suggesting a possible involvement in spliceosome regulation and in the synthesis of microRNAs with antitumor effects [128].

### 6.3. Clinical Relevance and Therapeutic Applications of Vitamin D in Colon Cancer

Available evidence suggests that vitamin D and its non-hypercalcemic analogues could be used in the prevention or adjuvant treatment of colorectal cancer, particularly in early stages or in patients with high VDR expression. Conversely, the presence of high levels of SNAIL1/SNAIL2 could be a marker of resistance to this therapy, which would be clinically useful in patient selection [129].

It has been observed that VDR expression in tumor stromal fibroblasts predicts a better clinical outcome, reinforcing the hypothesis that the protective effects of vitamin D are not limited to cancerous epithelial cells [130]. Complementarily, it has been shown that calcitriol and the Wnt3A factor exert additive and partially overlapping modulatory effects on the gene expression and behavior of human colon fibroblasts, regulating their proliferation, migration, and activation, thus contributing to the maintenance of intestinal homeostasis and influencing the progression of colorectal cancer [131].

Vitamin D regulates the behavior of colon stem cells in organoids derived from patients, showing differentiating effects on tumor cells and maintaining homeostasis on normal colonic stem cells [132]. In this regard, calcitriol has been shown to counteract aberrant differentiation induced by Notch pathway inhibition and BMP4 signaling activation, reinforcing its role as a key regulator of the balance between proliferation and differentiation in the intestinal epithelium [133]. Finally, vitamin D-induced activation of SIRT1 has been described as an additional epigenetic pathway contributing to its antiproliferative effects [134].

## 7. Recent Advances in Research on Vitamin D Analogues, Their Metabolism, and Clinical Applications

In recent decades, growing interest in the cellular functions of vitamin D has driven the development of structural analogues of calcitriol and the study of factors that affect its efficacy and bioavailability. These advances have expanded the therapeutic potential of vitamin D in various clinical contexts.

### 7.1. Development of Calcitriol Analogues: Structural Advances and Therapeutic Limitations

The synthesis of calcitriol analogues with blocked side chains has led to compounds with high vitamin D receptor (VDR) activation capacity, even higher than the natural ligand, in colon cancer cells [135]. Along these lines, non-steroidal fluorinated analogs of calcitriol such as CD578, WU515, and WY1113 have shown strong induction of cell differentiation, greater interaction with VDR coactivators, and repression of the β-catenin/TCF pathway, which is key in tumor processes [136].

Certain reviews focusing on these compounds highlight their biological efficacy, their ability to induce differentiation, repress oncogenic genes, and enhance the activity of the VDR-coactivator complex, positioning them as promising alternatives in oncology [137]. Similarly, analogues with side chains in unconventional positions such as C12 and C17, although with lower affinity for the VDR, have demonstrated antiproliferative effects and lower calcium toxicity in animal models, broadening their therapeutic window [138].

However, the clinical application of these analogues still faces limitations. Many of them show efficacy in preclinical models but require supraphysiological doses to achieve therapeutic effects, which increases the risk of hypercalcemia. This toxicity, linked to the high affinity of VDR for calcitriol, underscores the need to design safer analogues without compromising their efficacy [139].

### 7.2. Factors Associated with Vitamin D Deficiency

Various factors influence vitamin D levels, particularly in early life. It has been observed that infants who are breastfed for prolonged periods without supplementation have lower levels of 25(OH)D, a trend that is reversed with supplementation, supporting current recommendations [140].

It has also been shown that environmental pollutants such as heavy metals, endocrine disruptors, tobacco, and pollution affect the synthesis, activation, and absorption of vitamin D, potentially leading to deficiencies even in populations with good sun exposure or diet [141]. In particular, perfluoroalkyl compounds (PFAS), present in many everyday products, show high affinity for VDR in computer simulations. Some even exceed calcitriol in their binding capacity, suggesting a potential disruptive effect on endocrine, immune, and bone functions [142].

### 7.3. Therapeutic Applications of Vitamin D

From a public health perspective, the dose required to achieve adequate serum levels of vitamin D in adults has been evaluated. A recent meta-analysis in a healthy European population has determined that a daily intake of 25 μg (1000 IU) may be sufficient for 95% of individuals to achieve optimal concentrations of 25(OH)D. This inference has relevant implications for updating current dietary recommendations and preventing deficiencies at the population level [143].

Very recently, two epidemiologic research articles have revealed that baseeline vitamin D status with higher serum 25-hydroxyvitamin D levels are linked to a reduced risk of hepatocellular carcinoma, thus suggeting the potential role of 25-hydroxyvitamin D for the primary prevention of this oncologic disease [144,145].

Finally, the interaction between the gut microbiota, vitamin D, and its receptor has been positioned as a central physiological axis in the context of inflammatory bowel diseases (IBD). Both vitamin D and VDR contribute to immune balance and protection of the intestinal mucosa. In this regard, it has been proposed that combining probiotics with strategies to enhance VDR signaling could constitute an innovative therapeutic approach in pathologies such as Crohn’s disease or ulcerative colitis [146].

Taken together, these findings underscore the need for a multidimensional approach to the study and clinical application of vitamin D, considering both the molecular design of new analogues and the influence of external variables on its metabolism and efficacy.

## 8. Final Discussion

There are significant limitations in the available scientific literature on vitamin D. Many of the studies reviewed in the present work are observational in design, which prevents the establishment of firm causal relationships. Furthermore, significant heterogeneity has been identified between studies in terms of supplementation doses, study populations, and methods used to measure serum 25(OH)D levels, making it difficult to compare results and formulate consistent clinical recommendations. In oncology, for example, many vitamin D analogues have shown efficacy in vitro, but their therapeutic effects require supraphysiological doses that can induce hypercalcemia, limiting their clinical application [139].

Furthermore, environmental and metabolic factors that interfere with vitamin D synthesis and metabolism—such as exposure to pollutants, obesity, or altered gut microbiota—have not always been considered in depth in the studies reviewed, representing a potential bias that could affect the interpretation of results [102,141].

The limitations identified in the selected literature are reflected in the content of this review and contribute to its own limitations. It is necessary to continue advancing toward more robust, homogeneous, and controlled intervention studies that will allow for a more precise understanding of the preventive and therapeutic role of vitamin D in various pathologies, as well as optimize supplementation strategies in different populations. Since we have been guided by a systematic methodology but the final contents of the present review are more like those of a narrative review, it is also recommended to carried out formal systematic reviews on the topic to warrant higher-level evidence and quantitative synthesis.

## 9. Conclusions

The evidence gathered in the present work supports the idea that vitamin D has preventive potential in multiple diseases, including inflammatory, autoimmune, metabolic, and neurodegenerative disorders. Furthermore, supplementation strategies—when tailored to individual needs and contextual factors—can contribute significantly to maintaining optimal plasma concentrations, opening new avenues for their implementation in public health and preventive medicine.

In short, this work has served as a starting point for exploring the preventive and therapeutic potential of vitamin D from a multidisciplinary perspective, opening up new possibilities for research in the fields of public health, preventive medicine, and translational biomedicine.

## Figures and Tables

**Table 1:** Inclusion and exclusion criteria applied to the reading of the titles.

Inclusion Criteria	Exclusion Criteria
Articles relevant to this review	Articles not relevant to this review
Articles that, referring to the human species, are based on diverse and qualitatively and quantitatively representative population samples	Articles that, referring to the human species, focus on a specific population or an isolated case
Articles with unique information in their titles	Equivalent articles with slight spelling variations in the title
Articles that formally and concretely show the content of the document	Articles with titles that have a certain popular science character
Articles that approach the focus of the work (preventive and informative focus)	Articles that deviate from the focus of the work
Studies conducted in model organisms	Studies carried out in organisms other than those commonly used as study models
Articles in which the title reflects a biological approach (especially biochemical or physiological)	Articles that do not present a biological focus in the title (such as economic or chemical)
Titles with statements that relate to compelling results and discussions	Dubious titles that do not provide reliable ideas (either due to their content or the way they are expressed)

**Table 2:** Inclusion and exclusion criteria applied to the reading of the abstracts.

Inclusion Criteria	Exclusion Criteria
Articles that present an Abstract on which the criteria can be applied	Articles that do not present an Abstract on which the criteria can be applied
Articles relevant to this review	Articles not relevant to this review
Articles that approach the focus of the work (preventive and informative focus)	Articles that deviate from the focus of the work
Articles in which the title reflects a biological approach (especially biochemical or physiological)	Articles that do not present a biological focus in the title
Articles whose Abstract reflects in a concise, explanatory, organized and formal/objective manner all the contents of the summarized article	Articles whose Abstract presents a certain informative character or tone, does not present the information in an accessible way so that the reader can get a general idea and avoids the inclusion of some of the contents of the document
Articles whose Abstract shows statements that relate to compelling results and discussions, always with scientific rigor (indicating, for example, the extent to which the research has been carried out)	Articles whose Abstract is dubious and does not provide reliable ideas (either due to its content or its way of expressing it)
Articles where the information appearing in the title and Abstract is connected or interrelated, that is, the information contained in the title reflects the contents of the Abstract in a more concise manner.	Articles where the information appearing in the title and Abstract is not connected or interrelated, that is, where appropriate expressions are not used in the title to reflect the key idea intended to be conveyed by the Abstract.
Articles that, referring to the human species, are based on diverse population samples (and indicate the characteristics of each group) and are qualitatively and quantitatively representative, mainly in vivo studies.	Articles that, referring to the human species, focus on a specific population or an isolated case, as well as those that do not explicitly indicate the characteristics of the groups or any data of interest related to the origin of the population sample on which the study was carried out.
Studies conducted in model organisms	Studies carried out in organisms other than those commonly used as study models
Articles whose Abstract reflects the statistical results of the research	Articles whose Abstract does not reflect the statistical results of the research

**Table 3:** Inclusion and exclusion criteria applied to the reading of the complete articles.

Inclusion Criteria	Exclusion Criteria
Consistency between the abstract and the full text in terms of sample size and diversity	Inconsistencies between the abstract and the full text, especially regarding the sample
Original studies with adequate sample size and relevant population diversity (age, sex, origin, etc.)	Original studies with insufficient samples or without representative diversity
Reviews with a sufficient number of studies and evidence based on solid samples	Reviews that include few studies or are based on weak evidence (small or poorly defined samples)
Studies in animal models with a minimum sample size justified or accepted by field standards	Studies in animal models with very small sample sizes or without methodological justification
Clear description of key methodological aspects (sample size, inclusion criteria, analysis performed)	Lack of key information on methodology that prevents the quality of the study from being assessed

## Data Availability

No new data were created.

## Supplementary Materials

The following supporting information can be downloaded at: https://www.mdpi.com/article/10.3390/biomedicines13112733/s1, File S1: Blind Search Syntax.

## Abbreviations

The following abbreviations are used in this manuscript:

ASD	Autism Spectrum Disorder
EGF	Epithelial Growth Factor
EMT	Epithelial–Mesenchymal Transition
FGFR	Fibroblast Growth Factor Receptor
HT	Hashimoto’s Thyroiditis
IBD	Inflammatory Bowel Disease
IFN	Interferon
IL	Interleukin
MS	Multiple Sclerosis
NAFLD	Non-Alcoholic Fatty Liver Disease
ORCID	Open Researcher and Contributor ID
PCOS	Polycystic Ovary Syndrome
PDGFR	Platelet-Derived Growth Factor Receptor
PTH	Parathyroid Hormone
TFG	Transforming Growth Factor
TNF	Tumor Necrosis Factor
VDR	Vitamin D receptor
