# Supplementary material for: Vitamin D as a Systemic Regulatory Axis: From Homeostasis to Multiorgan Disease
Source: Biomedicines. 2025 Nov 7;13(11):2733. doi: 10.3390/biomedicines13112733 (PMC12650529; doi:10.3390/biomedicines13112733)
Supplement: Supplementary file 1 [file biomedicines-13-02733-s001.zip › biomedicines-3897725-supplementary.pdf]

SEARCH IN *SCOPUS*.

1. ***Vitamin D* or derivatives for all the fields:**

("vitamin D" OR "vitamin D2" OR "ergocalciferol" OR "vitamin D3" OR "cholecalciferol" OR “calcifediol” OR "calcidiol" OR "25-hydroxycholecalciferol" OR "25-hydroxyvitamin D" OR "25(OH)D" OR "25-hydroxyvitamin D3" OR “25(OH)D3” OR "calcitriol" OR "1,25-dihydroxycholecalciferol" OR "1,25-dihydroxyvitamin D" OR "1,25(OH)2D" OR "hormone D" OR "25-hydroxyvitamin D total" OR "(25(OH)D total)" OR "prohormone D" OR "pre-vitamin D")

2. **Forcing the presence of the term vitamin D in title:**

TITLE(("vitamin D" OR "vitamin D2" OR "ergocalciferol" OR "vitamin D3" OR "cholecalciferol" OR "calcifediol" OR "calcidiol" OR "25-hydroxycholecalciferol" OR "25-hydroxyvitamin D" OR "25(OH)D" OR "25-hydroxyvitamin D3" OR "25(OH)D3" OR "calcitriol" OR "1,25-dihydroxycholecalciferol" OR "1,25-dihydroxyvitamin D" OR "1,25(OH)2D" OR "hormone D" OR "25-hydroxyvitamin D total" OR "(25(OH)D total)" OR "prohormone D" OR "pre-vitamin D"))

3. **Articles written in English or Spanish:**

TITLE("vitamin D" OR "vitamin D2" OR "ergocalciferol" OR "vitamin D3" OR "cholecalciferol" OR "calcifediol" OR "calcidiol" OR "25-hydroxycholecalciferol" OR "25-hydroxyvitamin D" OR "25(OH)D" OR "25-hydroxyvitamin D3" OR "25(OH)D3" OR "calcitriol" OR "1,25-dihydroxycholecalciferol" OR "1,25-dihydroxyvitamin D" OR "1,25(OH)2D" OR "hormone D" OR "25-hydroxyvitamin D total" OR "(25(OH)D total)" OR "prohormone D" OR "pre-vitamin D") AND (LIMIT-TO (LANGUAGE,"English") OR LIMIT-TO (LANGUAGE,"Spanish"))

4. **Bifurcation of the search according to two sets of keywords.**

4.1. Path 1 (*health* o *disease* as keywords):

TITLE(("vitamin D" OR "vitamin D2" OR "ergocalciferol" OR "vitamin D3" OR "cholecalciferol" OR "calcifediol" OR "calcidiol" OR "25-hydroxycholecalciferol" OR "25-hydroxyvitamin D" OR "25(OH)D" OR "25-hydroxyvitamin D3" OR "25(OH)D3" OR "calcitriol" OR "1,25-dihydroxycholecalciferol" OR "1,25-dihydroxyvitamin D" OR "1,25(OH)2D" OR "hormone D" OR "25-hydroxyvitamin D total" OR "(25(OH)D total)" OR "prohormone D" OR "pre-vitamin D")) AND (LIMIT-TO (LANGUAGE,"English") OR LIMIT-TO (LANGUAGE,"Spanish")) AND (TITLE ("health" OR "disease") OR ABS ("health" OR "disease"))

4.1.1. **Addition of keyword** (*homeostasis* or *imbalance*)**:**

TITLE(("vitamin D" OR "vitamin D2" OR "ergocalciferol" OR "vitamin D3" OR "cholecalciferol" OR "calcifediol" OR "calcidiol" OR "25-hydroxycholecalciferol" OR "25-hydroxyvitamin D" OR "25(OH)D" OR "25-hydroxyvitamin D3" OR "25(OH)D3" OR "calcitriol" OR "1,25-dihydroxycholecalciferol" OR "1,25-dihydroxyvitamin D" OR "1,25(OH)2D" OR "hormone D" OR "25-hydroxyvitamin D total" OR "(25(OH)D total)" OR "prohormone D" OR "pre-vitamin D")) AND (LIMIT-TO (LANGUAGE,"English") OR LIMIT-TO (LANGUAGE,"Spanish")) AND (TITLE ("health" OR "disease") OR ABS ("health" OR "disease")) AND (TITLE ("homeostasis" OR “imbalance”) OR ABS ("homeostasis" OR “imbalance”))

4.2. Path 2 (*supplementation* or *supplement** derivatives as keywords):

TITLE("vitamin D" OR "vitamin D2" OR "ergocalciferol" OR "vitamin D3" OR "cholecalciferol" OR "calcifediol" OR "calcidiol" OR "25-hydroxycholecalciferol" OR "25-hydroxyvitamin D" OR "25(OH)D" OR "25-hydroxyvitamin D3" OR "25(OH)D3" OR "calcitriol" OR "1,25-dihydroxycholecalciferol" OR "1,25-dihydroxyvitamin D" OR "1,25(OH)2D" OR "hormone D" OR "25-hydroxyvitamin D total" OR "(25(OH)D total)" OR "prohormone D" OR "pre-vitamin D") AND (LIMIT-TO (LANGUAGE,"English") OR LIMIT-TO (LANGUAGE,"Spanish")) AND (TITLE (“supplementation” OR “supplement*”) OR ABS (“supplementation” OR “supplement*”))

4.2.1. **Addition of keyword** (*serum levels*)**:**

TITLE("vitamin D" OR "vitamin D2" OR "ergocalciferol" OR "vitamin D3" OR "cholecalciferol" OR "calcifediol" OR "calcidiol" OR "25-hydroxycholecalciferol" OR "25-hydroxyvitamin D" OR "25(OH)D" OR "25-hydroxyvitamin D3" OR "25(OH)D3" OR "calcitriol" OR "1,25-dihydroxycholecalciferol" OR "1,25-dihydroxyvitamin D" OR "1,25(OH)2D" OR "hormone D" OR "25-hydroxyvitamin D total" OR "(25(OH)D total)" OR "prohormone D" OR "pre-vitamin D") AND (LIMIT-TO (LANGUAGE,"English") OR LIMIT-TO (LANGUAGE,"Spanish")) AND (TITLE (“supplementation” OR “supplement*”) OR ABS (“supplementation” OR “supplement*”)) AND (TITLE (“serum levels”) OR ABS (“serum levels”))

5. ***Peer review*ed articles, including reviews.**

5.1. Path 1:

TITLE(("vitamin D" OR "vitamin D2" OR "ergocalciferol" OR "vitamin D3" OR "cholecalciferol" OR "calcifediol" OR "calcidiol" OR "25-hydroxycholecalciferol" OR "25-hydroxyvitamin D" OR "25(OH)D" OR "25-hydroxyvitamin D3" OR "25(OH)D3" OR "calcitriol" OR "1,25-dihydroxycholecalciferol" OR "1,25-dihydroxyvitamin D" OR "1,25(OH)2D" OR "hormone D" OR "25-hydroxyvitamin D total" OR "(25(OH)D total)" OR "prohormone D" OR "pre-vitamin D")) AND (LIMIT-TO (LANGUAGE,"English") OR LIMIT-TO (LANGUAGE,"Spanish")) AND (TITLE ("health" OR "disease") OR ABS ("health" OR "disease")) AND (TITLE ("homeostasis" OR “imbalance”) OR ABS ("homeostasis" OR “imbalance”)) AND (LIMIT-TO (DOCTYPE , "ar") OR LIMIT-TO (DOCTYPE , "re"))

5.2. Path 2:

TITLE("vitamin D" OR "vitamin D2" OR "ergocalciferol" OR "vitamin D3" OR "cholecalciferol" OR "calcifediol" OR "calcidiol" OR "25-hydroxycholecalciferol" OR "25-hydroxyvitamin D" OR "25(OH)D" OR "25-hydroxyvitamin D3" OR "25(OH)D3" OR "calcitriol" OR "1,25-dihydroxycholecalciferol" OR "1,25-dihydroxyvitamin D" OR "1,25(OH)2D" OR "hormone D" OR "25-hydroxyvitamin D total" OR "(25(OH)D total)" OR "prohormone D" OR "pre-vitamin D") AND (LIMIT-TO (LANGUAGE,"English") OR LIMIT-TO (LANGUAGE,"Spanish")) AND (TITLE (“supplementation” OR “supplement*”) OR ABS (“supplementation” OR “supplement*”)) AND (TITLE (“serum levels”) OR ABS (“serum levels”)) AND (LIMIT-TO (DOCTYPE , "ar") OR LIMIT-TO (DOCTYPE , "re"))

SEARCH IN *PUBMED*.

1. ***Vitamin D* or derivatives for all the fields:**

("vitamin D" OR "vitamin D2" OR "ergocalciferol" OR "vitamin D3" OR "cholecalciferol" OR “calcifediol” OR "calcidiol" OR "25-hydroxycholecalciferol" OR "25-hydroxyvitamin D" OR "25(OH)D" OR "25-hydroxyvitamin D3" OR “25(OH)D3” OR "calcitriol" OR "1,25-dihydroxycholecalciferol" OR "1,25-dihydroxyvitamin D" OR "1,25(OH)2D" OR "hormone D" OR "25-hydroxyvitamin D total" OR "(25(OH)D total)" OR "prohormone D" OR "pre-vitamin D")

2. ***Vitamin D* or derivatives for all the fields:**

("vitamin D"[Title] OR "vitamin D2"[Title] OR "ergocalciferol"[Title] OR "vitamin D3"[Title] OR "cholecalciferol"[Title] OR "calcifediol"[Title] OR "calcidiol"[Title] OR "25-hydroxycholecalciferol"[Title] OR "25-hydroxyvitamin D"[Title] OR "25(OH)D"[Title] OR "25-hydroxyvitamin D3"[Title] OR "25(OH)D3"[Title] OR "calcitriol"[Title] OR "1,25-dihydroxycholecalciferol"[Title] OR "1,25-dihydroxyvitamin D"[Title] OR "1,25(OH)2D"[Title] OR "hormone D"[Title] OR "25-hydroxyvitamin D total"[Title] OR "(25(OH)D total)"[Title] OR "prohormone D"[Title] OR "pre-vitamin D"[Title])

3. **Articles written in English or Spanish:**

("vitamin D"[Title] OR "vitamin D2"[Title] OR "ergocalciferol"[Title] OR "vitamin D3"[Title] OR "cholecalciferol"[Title] OR "calcifediol"[Title] OR "calcidiol"[Title] OR "25-hydroxycholecalciferol"[Title] OR "25-hydroxyvitamin D"[Title] OR "25(OH)D"[Title] OR "25-hydroxyvitamin D3"[Title] OR "25(OH)D3"[Title] OR "calcitriol"[Title] OR "1,25-dihydroxycholecalciferol"[Title] OR "1,25-dihydroxyvitamin D"[Title] OR "1,25(OH)2D"[Title] OR "hormone D"[Title] OR "25-hydroxyvitamin D total"[Title] OR "(25(OH)D total)"[Title] OR "prohormone D"[Title] OR "pre-vitamin D"[Title]) AND ("english"[Language] OR "spanish"[Language])

4. **Bifurcation of the search according to two sets of keywords.**

4.1. Path 1 (*health* o *disease* as keywords):

("vitamin D"[Title] OR "vitamin D2"[Title] OR "ergocalciferol"[Title] OR "vitamin D3"[Title] OR "cholecalciferol"[Title] OR "calcifediol"[Title] OR "calcidiol"[Title] OR "25-hydroxycholecalciferol"[Title] OR "25-hydroxyvitamin D"[Title] OR "25(OH)D"[Title] OR "25-hydroxyvitamin D3"[Title] OR "25(OH)D3"[Title] OR "calcitriol"[Title] OR "1,25-dihydroxycholecalciferol"[Title] OR "1,25-dihydroxyvitamin D"[Title] OR "1,25(OH)2D"[Title] OR "hormone D"[Title] OR "25-hydroxyvitamin D total"[Title] OR "(25(OH)D total)"[Title] OR "prohormone D"[Title] OR "pre-vitamin D"[Title]) AND ("english"[Language] OR

"spanish"[Language]) AND ("health"[Title/Abstract] OR "disease"[Title/Abstract])

4.1.1. Addition of keyword (*homeostasis* or *imbalance*)**:**

("vitamin D"[Title] OR "vitamin D2"[Title] OR "ergocalciferol"[Title] OR "vitamin D3"[Title] OR "cholecalciferol"[Title] OR "calcifediol"[Title] OR "calcidiol"[Title] OR "25-hydroxycholecalciferol"[Title] OR "25-hydroxyvitamin D"[Title] OR "25(OH)D"[Title] OR "25-hydroxyvitamin D3"[Title] OR "25(OH)D3"[Title] OR "calcitriol"[Title] OR "1,25-dihydroxycholecalciferol"[Title] OR "1,25-dihydroxyvitamin D"[Title] OR "1,25(OH)2D"[Title] OR "hormone D"[Title] OR "25-hydroxyvitamin D total"[Title] OR "(25(OH)D total)"[Title] OR "prohormone D"[Title] OR "pre-vitamin D"[Title]) AND ("english"[Language] OR "spanish"[Language]) AND ("health"[Title/Abstract] OR "disease"[Title/Abstract]) AND ("homeostasis"[Title/Abstract] OR “imbalance”[Title/Abstract])

4.2. Path 2 (*supplementation* or *supplement** derivatives as keywords):

("vitamin D"[Title] OR "vitamin D2"[Title] OR "ergocalciferol"[Title] OR "vitamin D3"[Title] OR "cholecalciferol"[Title] OR "calcifediol"[Title] OR "calcidiol"[Title] OR "25-hydroxycholecalciferol"[Title] OR "25-hydroxyvitamin D"[Title] OR "25(OH)D"[Title] OR "25-hydroxyvitamin D3"[Title] OR "25(OH)D3"[Title] OR "calcitriol"[Title] OR "1,25-dihydroxycholecalciferol"[Title] OR "1,25-dihydroxyvitamin D"[Title] OR "1,25(OH)2D"[Title] OR "hormone D"[Title] OR "25-hydroxyvitamin D total"[Title] OR "(25(OH)D total)"[Title] OR "prohormone D"[Title] OR "pre-vitamin D"[Title]) AND ("english"[Language] OR "spanish"[Language]) AND ("supplementation"[Title/Abstract] OR “supplement*”[Title/Abstract])

4.2.1. Addition of keyword (*serum levels*)**:**

("vitamin D"[Title] OR "vitamin D2"[Title] OR "ergocalciferol"[Title] OR "vitamin D3"[Title] OR "cholecalciferol"[Title] OR "calcifediol"[Title] OR "calcidiol"[Title] OR "25-hydroxycholecalciferol"[Title] OR "25-hydroxyvitamin D"[Title] OR "25(OH)D"[Title] OR "25-hydroxyvitamin D3"[Title] OR "25(OH)D3"[Title] OR "calcitriol"[Title] OR "1,25-dihydroxycholecalciferol"[Title] OR "1,25-dihydroxyvitamin D"[Title] OR "1,25(OH)2D"[Title] OR "hormone D"[Title] OR "25-hydroxyvitamin D total"[Title] OR "(25(OH)D total)"[Title] OR "prohormone D"[Title] OR "pre-vitamin D"[Title]) AND ("english"[Language] OR "spanish"[Language]) AND ("supplementation"[Title/Abstract] OR “supplement*”[Title/Abstract]) AND ("serum levels"[Title/Abstract])

SEARCH IN *WEB OF SCIENCE*.

1. *Vitamin D* or derivatives for all the fields:

TS=(("vitamin D" OR "vitamin D2" OR "ergocalciferol" OR "vitamin D3" OR "cholecalciferol" OR “calcifediol” OR "calcidiol" OR "25-hydroxycholecalciferol" OR "25-hydroxyvitamin D" OR "25(OH)D" OR "25-hydroxyvitamin D3" OR “25(OH)D3” OR "calcitriol" OR "1,25-dihydroxycholecalciferol" OR "1,25-dihydroxyvitamin D" OR "1,25(OH)2D" OR "hormone D" OR "25-hydroxyvitamin D total" OR "(25(OH)D total)" OR "prohormone D" OR "pre-vitamin D"))

2. *Vitamin D* or derivatives for all the fields:

TI=(("vitamin D" OR "vitamin D2" OR "ergocalciferol" OR "vitamin D3" OR "cholecalciferol" OR “calcifediol” OR "calcidiol" OR "25-hydroxycholecalciferol" OR "25-hydroxyvitamin D" OR "25(OH)D" OR "25-hydroxyvitamin D3" OR “25(OH)D3” OR "calcitriol" OR "1,25-dihydroxycholecalciferol" OR "1,25-dihydroxyvitamin D" OR "1,25(OH)2D" OR "hormone D" OR "25-hydroxyvitamin D total" OR "(25(OH)D total)" OR "prohormone D" OR "pre-vitamin D"))

3. Articles written in English or Spanish:

TI=("vitamin D" OR "vitamin D2" OR "ergocalciferol" OR "vitamin D3" OR "cholecalciferol" OR “calcifediol” OR "calcidiol" OR "25-hydroxycholecalciferol" OR "25-hydroxyvitamin D" OR "25(OH)D" OR "25-hydroxyvitamin D3" OR “25(OH)D3” OR "calcitriol" OR "1,25-dihydroxycholecalciferol" OR "1,25-dihydroxyvitamin D" OR "1,25(OH)2D" OR "hormone D" OR "25-hydroxyvitamin D total" OR "(25(OH)D total)" OR "prohormone D" OR "pre-vitamin D") AND LA=(“English” OR “Spanish”)

4. Bifurcation of the search according to two sets of keywords.

4.1. Path 1 (*health* o *disease* as keywords):

TI=("vitamin D" OR "vitamin D2" OR "ergocalciferol" OR "vitamin D3" OR "cholecalciferol" OR “calcifediol” OR "calcidiol" OR "25-hydroxycholecalciferol" OR "25-hydroxyvitamin D" OR "25(OH)D" OR "25-hydroxyvitamin D3" OR “25(OH)D3” OR "calcitriol" OR "1,25-dihydroxycholecalciferol" OR "1,25-dihydroxyvitamin D" OR "1,25(OH)2D" OR "hormone D" OR "25-hydroxyvitamin D total" OR "(25(OH)D total)" OR "prohormone D" OR "pre-vitamin D") AND LA=(“English” OR “Spanish”) AND (TI=(“health” OR “disease") OR AB=(“health” OR “disease”))

4.1.1. **Addition of keyword** (*homeostasis* or *imbalance*)**:**

TI=("vitamin D" OR "vitamin D2" OR "ergocalciferol" OR "vitamin D3" OR "cholecalciferol" OR “calcifediol” OR "calcidiol" OR "25-hydroxycholecalciferol" OR "25-hydroxyvitamin D" OR "25(OH)D" OR "25-hydroxyvitamin D3" OR “25(OH)D3” OR "calcitriol" OR "1,25-dihydroxycholecalciferol" OR "1,25-dihydroxyvitamin D" OR "1,25(OH)2D" OR "hormone D" OR "25-hydroxyvitamin D total" OR "(25(OH)D total)" OR "prohormone D" OR "pre-vitamin D") AND LA=(“English” OR “Spanish”) AND (TI=(“health” OR “disease") OR AB=(“health” OR “disease”)) AND (TI=(“homeostasis” OR “imbalance”) OR AB=(“homeostasis” OR “imbalance”))

5. Path 2 (*supplementation* or *supplement** derivatives as keywords):):

TI=("vitamin D" OR "vitamin D2" OR "ergocalciferol" OR "vitamin D3" OR "cholecalciferol" OR “calcifediol” OR "calcidiol" OR "25-hydroxycholecalciferol" OR "25-hydroxyvitamin D" OR "25(OH)D" OR "25-hydroxyvitamin D3" OR “25(OH)D3” OR "calcitriol" OR "1,25-dihydroxycholecalciferol" OR "1,25-dihydroxyvitamin D" OR "1,25(OH)2D" OR "hormone D" OR "25-hydroxyvitamin D total" OR "(25(OH)D total)" OR "prohormone D" OR "pre-vitamin D") AND LA=(“English” OR “Spanish”) AND (TI=(“ supplementation” OR “supplement*") OR AB=(“ supplementation” OR “supplement*"))

5.1. Addition of keyword (*serum levels*)**:**

TI=("vitamin D" OR "vitamin D2" OR "ergocalciferol" OR "vitamin D3" OR "cholecalciferol" OR “calcifediol” OR "calcidiol" OR "25-hydroxycholecalciferol" OR "25-hydroxyvitamin D" OR "25(OH)D" OR "25-hydroxyvitamin D3" OR “25(OH)D3” OR "calcitriol" OR "1,25-dihydroxycholecalciferol" OR "1,25-dihydroxyvitamin D" OR "1,25(OH)2D" OR "hormone D" OR "25-hydroxyvitamin D total" OR "(25(OH)D total)" OR "prohormone D" OR "pre-vitamin D") AND LA=(“English” OR “Spanish”) AND (TI=(“ supplementation” OR “supplement*") OR AB=(“ supplementation” OR “supplement*")) AND (TI=(“serum levels”) OR AB=(“serum levels”))

6. *Peer reviewe*d articles, including reviews.

6.1. Path 1:

TI=("vitamin D" OR "vitamin D2" OR "ergocalciferol" OR "vitamin D3" OR "cholecalciferol" OR “calcifediol” OR "calcidiol" OR "25-hydroxycholecalciferol" OR "25-hydroxyvitamin D" OR "25(OH)D" OR "25-hydroxyvitamin D3" OR “25(OH)D3” OR "calcitriol" OR "1,25-dihydroxycholecalciferol" OR "1,25-dihydroxyvitamin D" OR "1,25(OH)2D" OR "hormone D" OR "25-hydroxyvitamin D total" OR "(25(OH)D total)" OR "prohormone D" OR "pre-vitamin D") AND LA=(“English” OR “Spanish”) AND (TI=(“health” OR “disease") OR AB=(“health” OR “disease”)) AND (TI=(“homeostasis” OR “imbalance”) OR AB=(“homeostasis” OR “imbalance”)) AND (DT=("Article" OR "Review"))

6.2. Path 2:

TI=("vitamin D" OR "vitamin D2" OR "ergocalciferol" OR "vitamin D3" OR "cholecalciferol" OR “calcifediol” OR "calcidiol" OR "25-hydroxycholecalciferol" OR "25-hydroxyvitamin D" OR "25(OH)D" OR "25-hydroxyvitamin D3" OR “25(OH)D3” OR "calcitriol" OR "1,25-dihydroxycholecalciferol" OR "1,25-dihydroxyvitamin D" OR "1,25(OH)2D" OR "hormone D" OR "25-hydroxyvitamin D total" OR "(25(OH)D total)" OR "prohormone D" OR "pre-vitamin D") AND LA=(“English” OR “Spanish”) AND (TI=(“ supplementation” OR “supplement*") OR AB=(“ supplementation” OR “supplement*")) AND (TI=(“serum levels”) OR AB=(“serum levels”)) AND (DT=("Article" OR "Review"))
